# An Anthropogenic Habitat Facilitates the Establishment of Non-Native Birds by Providing Underexploited Resources

**DOI:** 10.1371/journal.pone.0135833

**Published:** 2015-08-14

**Authors:** Martin J. P. Sullivan, Richard G. Davies, Hannah L. Mossman, Aldina M. A. Franco

**Affiliations:** 1 School of Environmental Sciences, University of East Anglia, Norwich Research Park, Norwich, NR4 7TY, United Kingdom; 2 School of Geography, University of Leeds, Leeds, LS2 9JT, United Kingdom; 3 School of Biological Sciences, University of East Anglia, Norwich Research Park, Norwich, NR4 7TY, United Kingdom; 4 School of Science and the Environment, Manchester Metropolitan University, Chester Street, Manchester, M1 5GD, United Kingdom; Liverpool John Moores University, UNITED KINGDOM

## Abstract

Anthropogenic modification of habitats may reduce the resources available for native species, leading to population declines and extinction. These same habitats often have the highest richness of non-native species. This pattern may be explained if recently human-modified habitats provide novel resources that are more accessible to non-native species than native species. Using non-native birds in the Iberian Peninsula as a case study, we conduct a large-scale study to investigate whether non-native species are positively associated with human modified habitats, and to investigate whether this positive association may be driven by the presence of resources that are not fully exploited by native species. We do this by comparing the functional diversity and resource use of native and non-native bird communities in a recently human-modified habitat (rice fields) and in more traditional habitats in the Iberian Peninsula. The functional diversity of native bird communities was lower in rice fields, but non-native birds were positively associated with rice fields and plugged this gap. Differences in resource use between native and non-native species allowed non-native species to exploit resources that were plentiful in rice fields, supporting the role of underexploited resources in driving the positive association of non-native birds with rice fields. Our results provide a potential mechanism explaining the positive association of non-native species with anthropogenic habitats, and further work is needed to test if this applies more generally.

## Introduction

One of the most fundamental questions in ecology is the extent to which communities are saturated with species [1, 2]. The transport and introduction of non-native species to new areas has provided a natural experiment to investigate this [3]. The ability of many non-native species to establish in communities without a corresponding extinction of native species demonstrates that communities can often accept additional species [4]. However, competition for resources with native species can influence their ability to become established [5]. For example, non-native plants that are distantly related to native species, and therefore likely to have different resource requirements, are more likely to establish successfully [6].

Experimental work has linked community invasibility to the amount of resources that are unexploited by native species [7], which can occur when resource pulses temporarily provide surplus resources [7] or spatial heterogeneity limits the ability of species to exploit all available resources [8]. Changes to the state of a system, such as alteration to fire regimes, can also lead to unexploited resources [9]. Such changes can act as an environmental filter [10], where a portion of the species found in the original community share traits that allow them to persist in the modified one. A consequence of environmental filtering is that functional traits (i.e. traits that influence how species interact with the environment) are lost from the community [11–13], potentially including traits that influence resource use. Such a reduction in the diversity of traits relating to resource use is likely to reduce the likelihood of species being able to utilise novel resources provided by the altered system. Small-scale experiments have shown that such loss of the diversity of functional traits (loss of functional diversity) can lower the resistance of communities to invasion [14], but it is unknown whether this happens at large spatial scales and in non-experimental ecosystems. The presence of unexploited resources may be brief, as species in the regional species pool able to exploit these novel resources could colonise the community [15]. However, if such species are absent from the regional species pool, then unexploited resources may be present for longer. We expect this to be likely when resources provided by the new system have few local analogues. Some human-modified landscapes potentially provide an example of this. For example, conversion of natural habitats to agriculture can dramatically change the resources available [16], leading to the non-random loss of species [17]. While some agricultural habitats, for example wood-pasture, contain functionally similar elements to those in natural habitats [18], others have no local analogues.

Human-modified landscapes often have high non-native species richness [19]. However, it is not known whether the presence of unexploited resources is a likely mechanism behind this pattern. The number of non-native species established in a given location depends on the number of species that can pass through sufficient stages in the invasion pathway to be recorded as having self-sustaining populations there [20], so factors aside from community invasibility affect non-native species richness [21, 22]. Establishing the mechanisms underlying high species richness in human-modified landscapes is therefore challenging.

Here we investigate whether the presence of unexploited resources in a recently created human-modified habitat (rice fields) could have facilitated species invasion by looking at non-native birds in the Iberian Peninsula, where the number of established non-native species has increased rapidly in recent decades [23]. Non-native birds in the Iberian Peninsula are positively associated with rice fields at both coarse and fine scales [23, 24]. At coarse scales the relationship could be confounded by factors such as climate and propagule pressure, but these cannot account for fine scale associations. Instead, fine scale associations may reflect selection of rice fields due to the resources they provide (i.e. environmental filtering) and/or reduced interspecific competition from native species due to the presence of unexploited resources. Rice fields are a fairly recent land-use in the Iberian Peninsula; despite some localised cultivation prior to the 20^th^ century, widespread cultivation has only occurred since the 1930s [25]. Unlike traditional agricultural systems, which comprise a mosaic of open habitats and wooded features, rice fields comprise open fields that are seasonally flooded, crossed by ditches with wetland vegetation bounded by field margins with forbs and rough grass. While the individual resources found in rice fields are present in other habitats (e.g. emergent vegetation is also found in natural wetlands), the combination of these resources in rice fields is potentially novel. We compare bird communities in rice fields (a recently human-modified habitat) and in more traditional land-uses containing habitat elements found in rice fields. With observational data we cannot directly test whether the patterns we observe result from interspecific competition or from environmental filtering. Instead, we investigate whether patterns are consistent with those expected if underexploited resources were facilitating the establishment of non-native species in rice fields. Specifically, we investigate whether resources in rice fields are not fully exploited by native species and whether differences in resource use for feeding and shelter allow non-native species to exploit these resources.

## Methods

### Field survey

We focus on four species of bird that have established non-native populations in the Iberian Peninsula. These are common waxbill *Estrilda astrild*, red avadavat *Amandava amandava*, yellow-crowned bishop *Euplectes afer* and black-headed weaver *Ploceous melanocephalus*, which are established in the wider countryside, so may have benefited from novel land-uses in these areas. Common waxbills became established in the Iberian Peninsula in the 1960s [24], red avadavats established before 1980, yellow-crowned bishops established in the mid-1980s, while black-headed weavers established in the 1990s [23]. These species are all seed-eating passerines, and are therefore functionally similar to native finches, sparrows and buntings.

**Fig 1:**
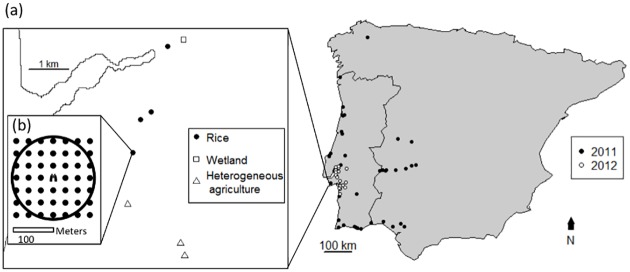
Location of survey sites and schematic of survey protocol. (a) Location of survey sites in the Iberian Peninsula. The centroids of each site are plotted. Sites surveyed in 2011 are shown by filled circles, and sites surveyed in 2012 are shown by open circles. The insert map shows the location of point counts at one site. Point counts in rice fields are shown by filled circles, point counts in wetlands are shown by open squares, and point counts in heterogeneous agriculture (defined as Corine land-cover level two class 24) are shown by open triangles. The remaining area not sampled by point counts is largely forestry. (b) Schematic of sampling protocol at each point count. The observer (position shown by binoculars) records birds seen within a 100m radius (shown by circle). Resources are recorded at regularly spaced points (shown by filled circles, resources also recorded at position of observer).

Surveys of these species were conducted in Portugal and western Spain in the breeding seasons (April to June) of 2011 and 2012. We surveyed seed-eating bird communities in rice fields and adjacent agriculture (arable and heterogenous mixtures of fruit crops and arable), rivers and natural wetlands. These adjacent open habitats represent more traditional land-uses and each contain some of the habitat elements found in rice fields; the emergent vegetation along ditches used to irrigate rice fields is also found along rivers and natural wetlands, while the mix of crops, field margins and more structural vegetation (e.g. emergent vegetation along ditches, or trees and bushes along field boundaries or interspersed with crops) is found in both rice fields and other agriculture. We selected 51 areas with both rice fields and more traditional land uses as well as 10 areas containing open habitats but lacking rice fields (Fig 1a). The latter sites enabled open habitats in landscapes lacking rice fields to be sampled. The extent of accessible target habitats varied between sites, so the number of point counts carried out varied between sites to reflect the limitations imposed by limited accessible habitat at some sites. Thus we carried out up to 16 point counts per site (mean = 7.5, sd = 3.3, range = 1–16, but with only one site with 1 point count), sampling rice fields and adjacent open habitats (see Fig 1a for example). A potential consequence of this unbalanced sampling between sites could be that spatial variation in abundance between sites, if correlated with variation in the number of points counts in rice fields, could enhance (or obscure) differences between rice fields and other habitats. To avoid this potential bias, we incorporated site as a random effect in subsequent analysis (see data analysis section). In 2011, sites were located across Portugal and western Spain, while in 2012 sites were located in the Tagus and Sado valleys of Portugal. Sites did not overlap, with the exception of two sites that were re-surveyed in 2012 to take advantage of improved access to the rice fields (point counts were never in the same location). Point counts within a site were surveyed within 1.1 ± 3.2 SD days of each other. Point counts lasted five minutes, and all seed-eating bird species seen within a 100m radius of the observer were recorded. For each individual or flock of target species seen, we recorded the distance from the observer (using a laser range finder, Bushnell, Kansas City USA), the activity they were doing (feeding, shelter—i.e. non-feeding rest activity—or other—e.g. dust-bathing, displaying from a song post), the resource (i.e. trees and shrubs, emergent vegetation, rough (i.e. ungrazed) grass and forbs) they were using, and the number of individuals in the group. The same observer performed all point counts, including recording resource availability. Access to field sites in the Paul de Tornada and Reserva Natural do Estuário do Tejo protected areas was provided by Associação PATO (Helder Cardoso) and Instituto da Conservação da Natureza e das Florestas (Vitor Encarnacão); remaining field sites were publically accessible.

We also quantified the resources present at point count locations. The main resources we considered in this study were the extent of vegetation types that we expected birds to use for feeding and shelter. Shelter resources were defined as resources used for resting and protection between intervals of feeding, but similar resources were likely to be used for nesting and roosting (although this was not quantified here), and had the potential to influence the ability of birds to access food resources [26]. We therefore quantified the extent of forbs and rough grass (both expected to be used for feeding, as they provide seeds) and emergent vegetation and trees and bushes (both expected to be used for shelter, as they are tall and dense vegetation features potentially giving birds safety from predators). In addition to these, which were recorded at every point count location, we measured finer scale resources related to food availability and accessibility at a subset of point count locations. These were the species richness and average weight holding capacity of grasses and forbs in field margins. Species rich field margins were expected to provide a greater diversity of resources, due to differences in seed size between plant species, and provide resources for longer, due to differences in timing of seed bearing between plant species. The ability of plants to stay upright when weight is applied to their seed heads (weight holding capacity) influences the ability of birds to access seeds, as relatively heavy birds can land and feed on the seed heads of plants that can hold relatively high weights, whereas only lighter bird species can access plants with lower weight holding capacities. We quantify the potential resources provided by a habitat, and not the actual availability following exploitation by birds.

The amount of forbs, rough grass, emergent vegetation and trees and bushes at each point count location was recorded at 30m intervals on a grid stretching 90m in each direction from the point count location (i.e. 49 resource recording points per point count, see Fig 1b for schematic), using a laser range finder to establish the recording grid. The other resources (grass and forb species richness and plant weight holding capacity) were quantified at a subset of point counts in the Tagus and Sado valleys. These were recorded in 0.25m^2^ quadrats randomly located in field margins in rice fields (34 quadrats at 23 point count locations) and in field margins and similar grassland features in adjacent open habitats (22 quadrats at 10 point count locations). Although these data were collected from a subset of the total number of point count locations, the results from this more detailed assessment of resource availability were consistent with the results from the coarser recording of feeding resource availability described above (see results). In each quadrat we counted the number of grass and forb species, and estimated the percentage cover of each species. We measured the weight holding capacity of seed bearing plants (three measurements per species per site where seed-heads present, mean = 6.9 measurements per species) by attaching weights to the base of the seed head, and recording how many weights were needed to cause the plant to droop to the ground. From this we obtained an average measure of weight holding capacity for each species, and combined this with estimates of percentage cover of each species in each quadrat to obtain a community weighted mean of weight holding capacity for each quadrat. We obtained data on mass of bird species from [27–31] to assess the ability of native and non-native species to access these resources.

It is important to note that we compared sampling units (point counts or quadrats) in rice fields with sampling units in other habitats. Heterogeneity across all sampling units is likely to be higher in other habitats than in rice fields simply because the former category embraces a larger number of habitat types. However, this does not affect the validity of our analysis, because we compared sampling units (point counts or quadrats) in rice fields with sampling units in other habitats (rather than comparing data summed across all sampling units). Any differences in within-sampling unit heterogeneity will result from genuine differences in the resource composition of habitats.

### Quantifying resource selection

We used Jacobs index to quantify, for each species, the selection of each feeding and shelter resource given the availability of each resource. Jacobs index was calculated for each species and resource as *J* = (*u*—*a*)/(*u* + *a*– 2*ua*), where *u* is the proportion of observations of an activity where a given resource was used and *a* is the availability of that resource in point counts where the species was recorded [32]. By accounting for resource availability we ensure that our calculations of resource selection are not influenced by differences in the availability of resources between areas, thus meaning they reflect resource selection rather than being confounded by the role of these resources in driving occupancy. Results using the availability of resources across all point counts, and thus implicitly incorporating the effect of each resource on occupancy, are presented in S1 Fig.

### Accounting for detectability

We used distance analysis to model the detectability of each bird species. Detectability models were constructed using the R package mrds [33], using a half-normal function to model the decline in detectability with distance from the observer. The amount of emergent vegetation and trees and bushes (i.e. proportion of resource recoding points containing these habitat classes) in each point count location were included as covariates (as these could obscure birds reducing detectability), as was the flock size of each group of birds. All simplifications of this global model were fitted, and the model with the lowest AIC was used to predict the detection probability of each species at each point count location. Detection probabilities could be calculated for all non-native species and for seven of the native species (six native species were recorded but too infrequently to fit detectability models).

The density of each species was calculated by dividing the total number of individuals counted during a point count by the detection probability for that point count location. We tested whether detectability of native and non-native species differed between rice fields and other habitats by modelling the predicted detection probability of each species in each point count as a function of whether or not it was a non-native species, whether or not the point count was in a rice field, and the interaction of these variables. Although there was some variability between species (see S1 Table for estimated detection probabilities for each species), overall there was a significant interaction between rice field status and whether a species was non-native, with native species having a higher probability of being detected in rice fields (*t* = 2.034, *P* = 0.0422). The direction of this effect meant that it would bias against us finding the results we report (see discussion for more), so does not affect the validity of our conclusions.

### Functional diversity metrics

We use functional diversity metrics to quantify how the diversity of traits relating to resource use varied among bird communities. Communities with lower functional diversity are likely to be able to exploit a more limited range of resources than more functionally diverse communities, so if native bird communities had lower functional diversity in rice fields this would be consistent with the presence of unexploited resources there.

We obtained species trait data from published literature, supplemented by field observations (details in S1 Appendix), to construct a trait matrix containing all native and established non-native species belonging to the seed-eating guild present in Portugal and western Spain (four non-native and 17 native species). Traits that related to a species’ use of resources for feeding and nesting were selected (Table 1). Gower distance was used to convert the trait matrix to a distance matrix, as it can handle a mix of continuous, ordinal and categorical data [34]. We calculated the functional diversity (FD) of communities following [35]. This method can generate FD values for communities of any size, but only uses presence-absence data; methods that incorporate abundance data exist [36], but can only be used on communities with three or more species, so would have required discarding potentially important species poor communities. To calculate FD we used the distance matrix to generate a dendrogram containing all seed-eating bird species, and calculated the FD of the bird community at each point count by dividing the total branch lengths of a dendrogram containing all species in the community by the total branch lengths of a dendrogram containing all target species. We used average linkage to generate a dendrogram as, compared to single and complete linkage, it gave the dendrogram with the highest cophenetic correlation with the distance matrix (c = 0.735). The observed values of FD were compared with values obtained by running null models where the species composition of communities was changed while maintaining species richness [35]. Following Mendez *et al*. [37] we used two null models, one randomly selecting species from the entire seed-eating guild, and one where random selection of species was weighted by the observed frequencies of species. Null models were run 1000 times. The standardised effect size of functional dispersion was calculated by subtracting the mean FD of null models from observed FD, then dividing this by the standard deviation of FD from null models. Values of greater than zero indicate functional over-dispersion, which has been suggested to result from competitive exclusion of similar species, and values less than zero indicate functional under-dispersion, which can indicate habitat filtering and greater packing of functionally similar species [12]. Communities were more under-dispersed when a random null model was used, possibly as a result of broad-scale habitat filtering, but otherwise results were consistent between null models. Therefore only results from the null model maintaining species frequency (FD_freq_) have been presented.

**Table 1:** Traits used to calculate functional diversity metrics. Species were given a score for each category of each trait according to rules given in S1 Appendix.

Trait group	Categories	Data type
Adult diet	Seeds, Green plants, Invertebrates	Ordinal
Nestling diet	Seeds, Green plants, Invertebrates	Ordinal
Feeding agility	Upside down, Vertical stem, Bent stem	Ordinal
Feeding height	Ground, Herb layer, Tree layer	Ordinal
Feeding habitat	Weeds, Cultivated, Trees	Ordinal
Food plants	Grasses, Composite	Ordinal
Morphology	Culmen length, Bill length-depth ratio, Tarsus length, Wing length, Body mass	Continuous
Nest location	Ground, dry low vegetation, wet low vegetation, Tree, Hole nester, Cliff nester	Ordinal
Nest height	Nest height	Ordinal
Nesting season	January, February, March, April, May, June, July, August, September, October, November, December	Ordinal

### Quantifying habitat associations

We compared the habitat associations of native species with the habitat associations of non-native species in their native range. Many habitats found in the native range were not found in the Iberian Peninsula, and vice versa, so it was necessary to position different habitats on environmental gradients in order to compare them. To do this, we quantified the strength of a species’ association along two environmental gradients (wet to dry habitats, and open to closed (forested) habitats), following the approach of Dolman et al. [38] to quantitatively code habitat associations from qualitative descriptions in literature. In the absence of quantitative data on species habitat associations, this method provides a more transparent and objective alternative to qualitative discussion of habitat associations. We acknowledge that, despite the use of rules to assist with coding, this approach remains slightly subjective.

The gradient from wet to dry habitats was divided into four categories (extensive wetland, linear or fragmented wetland, damp habitats and dry habitats) and the gradient from open to closed habitats was divided into six categories (low growing vegetation, matrix of low growing vegetation and taller non-woody vegetation, extensive taller non-woody vegetation, matrix of woody and non-woody vegetation dominated by the latter, matrix of woody and non-woody vegetation dominated by the former, and forests and woodland). These categories were chosen to adequately describe the variation in habitats along these gradients. We mapped habitat types onto these gradients based on the above definitions (see S2 Table). We consulted literature references on species’ habitat associations [27–31], and converted these qualitative descriptions into quantitative scores for association strength, where zero corresponded to no association with that habitat type, +1 to qualitative descriptions indicating the species was weakly associated with the habitat, and +2 to strongly associated. These habitat association scores were converted into an overall score for each position on the gradients by taking the maximum score across all habitats at that point. All coding was performed by one person to ensure consistent interpretation of sources. Scores for each species are given in S3 Table.

### Data analysis

We tested all models involving spatial data for residual spatial autocorrelation using Moran’s *I* tests. In all cases, significant residual spatial autocorrelation was found. We addressed this by fitting mixed effects models with site as a random effect. This assumes that observations within the same site are correlated while observations in different sites are independent, and removed significant residual spatial autocorrelation (Moran’s *I* test *P*-values >0.05) for all models.

All statistical analyses were performed in R [39], using the library lme4 [40] to fit mixed effects models. The significance of model terms was assessed using likelihood-ratio tests, where a model containing a given term was compared to a nested model lacking that term. Within this framework, we modelled various response variables of interest (e.g. species richness, density, functional diversity, plant species richness) as a function of whether the sampling location was a rice field. Likelihood-ratio tests thus compare the fit of a model with sampling location habitat (rice field or other) as an explanatory variable with an intercept only null model. We tested for differences in attributes between native and non-native species (e.g. body mass, resource selection) using Wilcoxon-Mann-Whitney tests.

## Results

Seventeen species (four non-native and 13 native) of seed-eating birds were recorded (i.e. four species in the source pool were not recorded). All four non-native species were found in a higher proportion of rice field point counts than point counts in adjacent open habitat; the same was true for only three native species (Table 2). However, differences in these proportions were fairly small for many species, indicating that non-native species were not restricted to rice fields and most widely recorded native species occurred in rice fields to an extent. The non-native common waxbill was the second most widely recorded species, being present in 176 of 456 point counts. House sparrow *Passer domesticus*, goldfinch *Carduelis carduelis* and serin *Serinus serinus* were the most widely recorded native species, all being present in > 160 point counts (Table 2).

**Table 2:** Study species recorded, and number of sites and point counts present.

Species	Scientific name	Native	Number of sites present	Number of point counts in rice fields present (% in parenthesis)	Number of points counts in other open habitats present (% in parenthesis)
**Black-headed weaver**	***Ploceous melanocephalus***	**No**	**8**	**11 (5.5)**	**8 (3.1)**
Bullfinch	*Pyrrhula pyrrhula*	Yes	1	0 (0.0)	1 (0.4)
Chaffinch	*Fringilla coelebs*	Yes	13	6 (3.0)	21 (8.2)
**Common waxbill**	***Estrilda astrild***	**No**	**56**	**82 (41.0)**	**94 (36.7)**
Corn bunting	*Emberiza calandra*	Yes	35	67 (33.5)	75 (29.3)
Goldfinch	*Carduelis carduelis*	Yes	54	69 (34.5)	96 (37.5)
Greenfinch	*Carduelis chloris*	Yes	42	43 (21.5)	83 (32.4)
Hawfinch	*Coccothraustes coccothraustes*	Yes	1	1 (0.5)	0 (0.0)
House sparrow	*Passer domesticus*	Yes	60	129 (64.5)	151 (59.0)
Linnet	*Carduelis cannabina*	Yes	44	38 (19.0)	50 (19.5)
**Red avadavat**	***Amandava amandava***	**No**	**11**	**13 (6.5)**	**8 (3.1)**
Reed bunting	*Emberiza schoeniclus*	Yes	2	1 (0.5)	3 (1.2)
Serin	*Serinus serinus*	Yes	50	56 (28.0)	106 (41.4)
Siskin	*Carduelis spinus*	Yes	1	0 (0.0)	2 (0.8)
Spanish sparrow	*Passer hispaniolensis*	Yes	6	1 (0.5)	9 (3.5)
Tree sparrow	*Passer montnus*	Yes	14	11 (5.5)	16 (6.3)
**Yellow-crowned bishop**	***Euplectes afer***	**No**	**21**	**54 (27.0)**	**13 (5.1)**
Total			61	200	256

The native rock sparrow *Petronia petronia*, common crossbill *Loxia curvirostra*, ortolan bunting *Emberiza hortulana* and yellowhammer *Emberiza citronella* were not recorded in any point counts. Non-native species are shown in bold. Percentages show the percentage of point counts in a habitat type in which a species was recorded.

**Fig 2:**
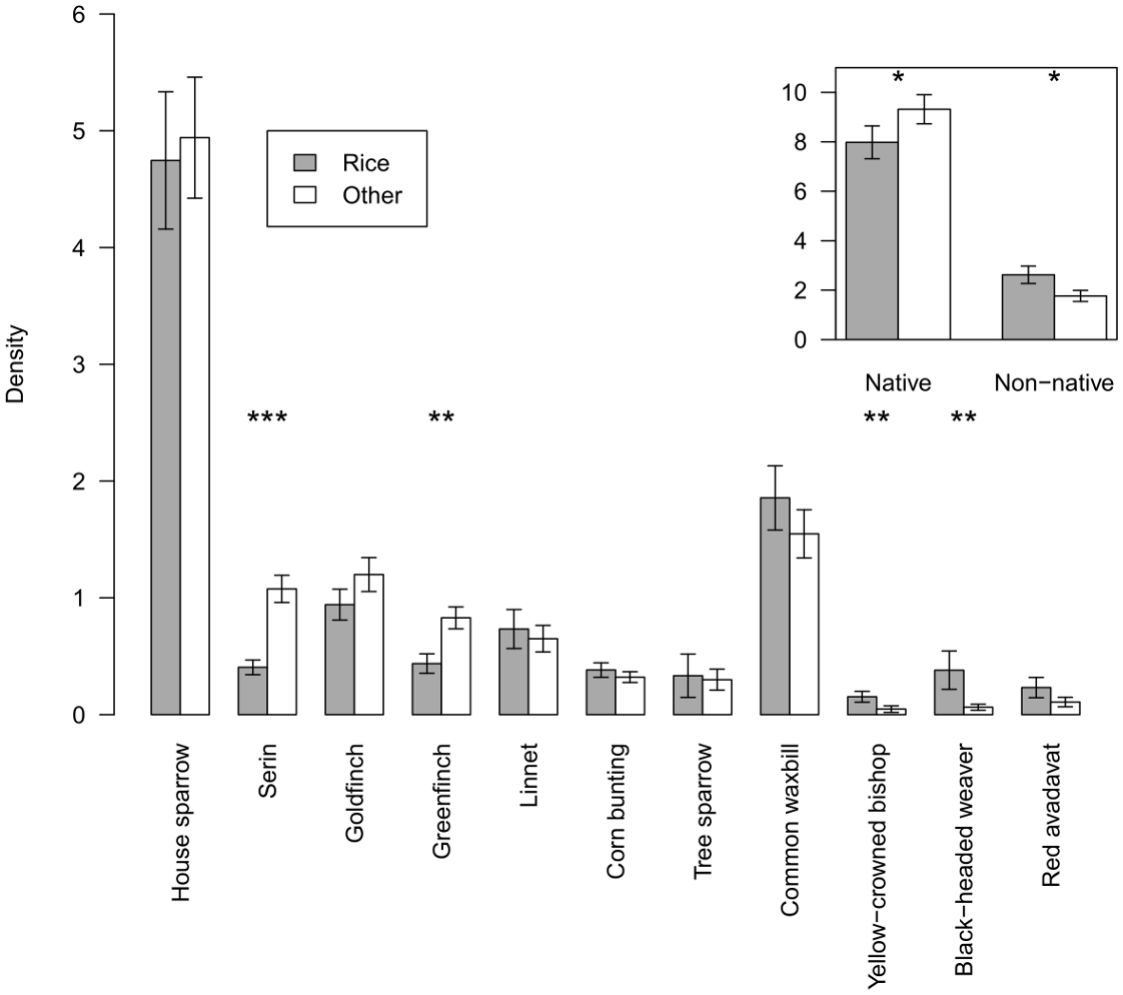
Density of native and non-native species in rice fields and adjacent open habitats. The main figure shows densities of each species, while the insert shows overall densities averaged across native and non-native species. Densities were calculated by dividing observed counts during point counts by estimated detection probabilities based on the habitat at each point count location.

The density of native species was lower in rice fields than in other habitats (χ^2^ = 3.951, *P* = 0.047, Fig 2), with significantly lower densities of serin *Serinus serinus* and greenfinch *Carduelis chloris* in rice fields (Fig 2). Native species richness was lower in rice fields, but not significantly so (χ^2^ = 3.137, *P* = 0.077). Both the density and species richness of non-native species was higher in rice fields than in other habitats (density: χ^2^ = 4.824, *P* = 0.028, species richness: χ^2^ = 7.626, *P* = 0.006, Fig 2). When densities of individual non-native species were looked at, all non-native species had higher densities in rice fields than other habitats, with statistically significant differences for yellow-crowned bishop and black-headed weaver (Fig 2).

**Fig 3:**
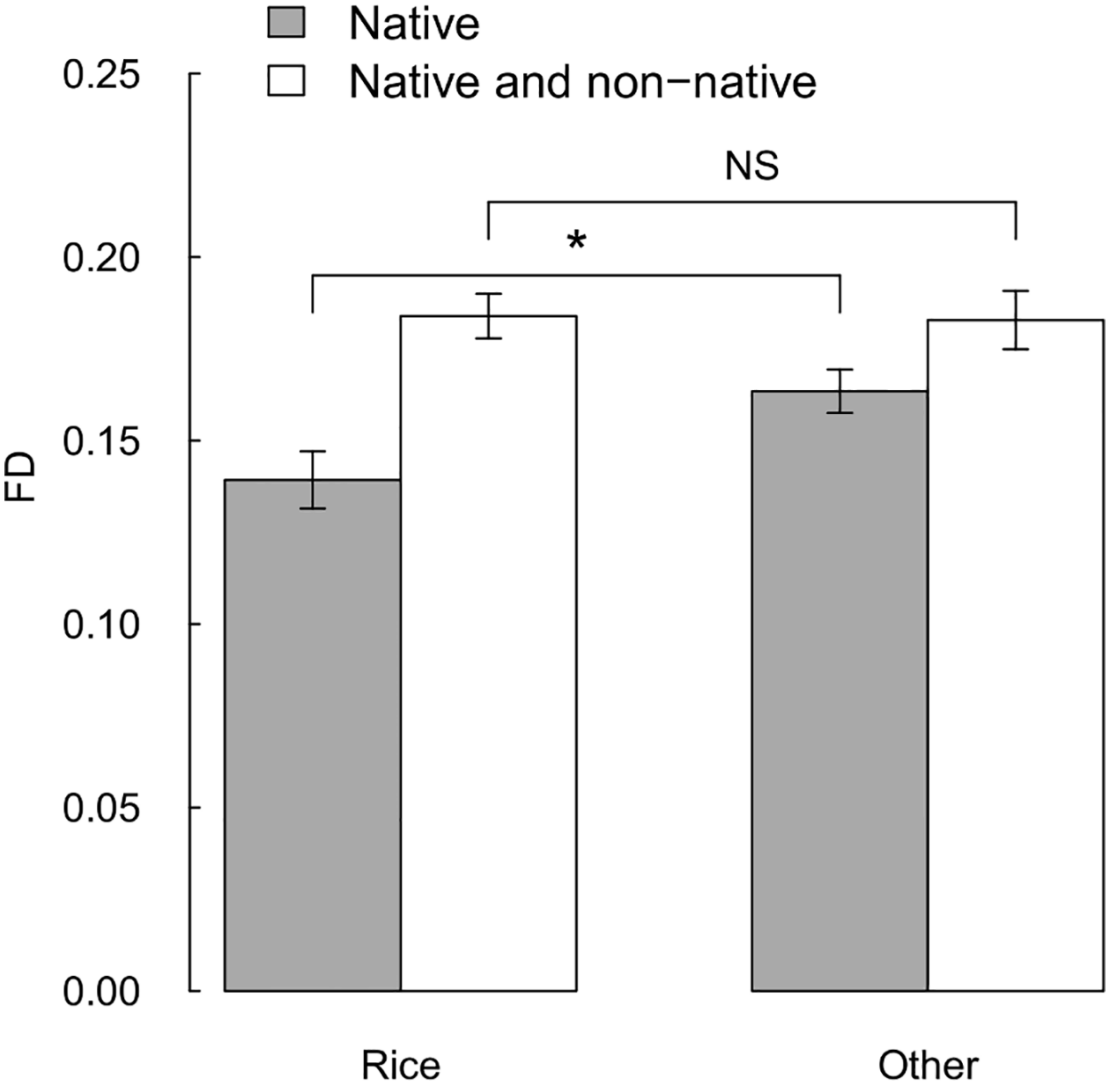
Functional diversity (FD) of native and native and non-native seed-eating bird communities in rice fields and other open habitats. Error bars show standard error. * denotes *P*<0.05, NS denotes *P*>0.05.

Native seed-eating bird communities had lower functional diversity in rice fields than in adjacent open habitats (χ^2^ = 4.329, *P* = 0.038, Fig 3). Non-native species plugged this gap, as the functional diversity of the combined native and non-native seed-eating bird community was not significantly different between rice fields and adjacent open habitats (χ^2^ = 0.011, *P* = 0.918, Fig 3). This was not driven by a single non-native species, as functional diversity remained not significantly different between rice fields and adjacent open habitats when the above analyses was repeated with one non-native species removed each time (*P* ≥ 0.340). Native bird communities were functionally random as FD_freq_ was not significantly different from zero (FD_freq_ = 0.02 ± 0.05, *t* = 0.33, *P* = 0.745), but bird communities in rice fields were more functionally dispersed in rice fields (Δ FD_freq_ = 0.25 ± 0.11, *P* = 0.024, S2 Appendix), indicating that in rice fields there was lower packing of functionally similar species. Functional dispersion was negatively correlated with species richness (*r* = -0.13, *P* = 0.017), indicating that species packing increased with species richness.

**Fig 4:**
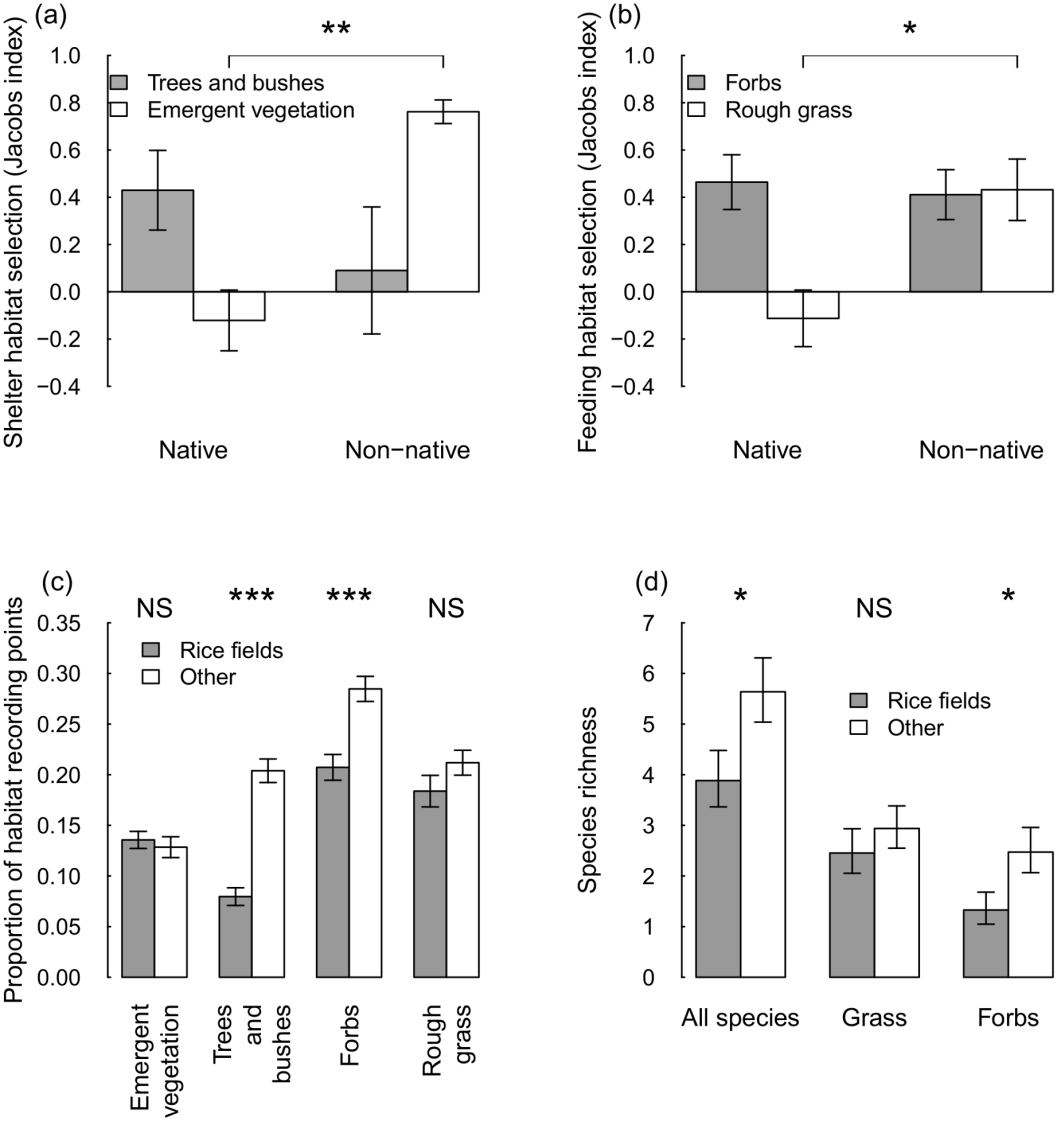
Availability and selection of resources by native and non-native species. Selection of resources for (a) shelter and (b) feeding by native and non-native species. Positive values of Jacobs index indicate that a habitat is selected more than expected given availability, and negative values indicate that it is selected less than expected. (c) Proportion of resource sampling points containing selected habitat types at point count locations in rice fields and other open habitats and (d) species richness of plants in rice field margins and adjacent grassland. Error bars show standard error. *** denotes *P*<0.001, ** denotes *P*<0.01, * denotes P<0.05, NS denotes *P*>0.05.

Differences in the selection of shelter resources could explain these results. Non-native species showed a greater preference for using emergent vegetation for shelter than native species (W = 24, *P* = 0.009, Fig 4a), while native species primarily used trees and bushes for shelter (Fig 4a). Trees and bushes were found less frequently in rice fields than adjacent open habitats (χ^2^ = 37.34, *P* < 0.001, Fig 4c), while there was no significant difference in the amount of emergent vegetation (χ^2^ = 0.276, *P* = 0.599, Fig 4c).

There were also differences in the amount of feeding resources provided by rice fields and adjacent open habitats. Both native and non-native species selected forbs for feeding, while non-native species showed a greater preference for rough grass (W = 18, *P* = 0.024, Fig 4b). There was no significant difference between the amount of rough grass in rice fields and other sites (χ^2^ = 1.525, *P* = 0.217) but more forbs were found in non-rice field sites (χ^2^ = 9.119, *P* = 0.003, Fig 4b).

Fine scale resources related to feeding also varied between rice fields and other open habitats. Plant species richness was lower in rice fields than in adjacent habitats with 1.5 ± 0.6 fewer species in rice field margins (χ^2^ = 5.750, *P* = 0.016, Fig 4d). Grass species richness did not significantly differ between rice field margins and adjacent grassland in non-rice field habitats (χ^2^ = 0.988, *P* = 0.320, Fig 4d), but forb species richness was significantly lower in rice field margins (χ^2^ = 5.640, *P* = 0.018, Fig 4d). Thus, grasses made up more of the available food resources in rice fields than in other open habitats. Plant weight holding capacity was 1.9g lower in rice fields than adjacent grasslands (mean weight holding capacity in rice fields = 2.6 ± 0.4g, mean weight holding capacity in other grasslands = 4.5 ± 0.3g, *W* = 215, *P* = 0.002). This indicates that proportionally more food resources in rice fields were only available to light bodied and/or agile species. Non-native species were, on average, 11.5g lighter than native species (non-native body mass = 15.4 ± 3.6g, native body mass = 26.9 ± 2.6g, *W* = 64, *P* = 0.035), with two non-native species (common waxbill and red avadavat) being lighter than any native seed-eating bird species.

**Fig 5:**
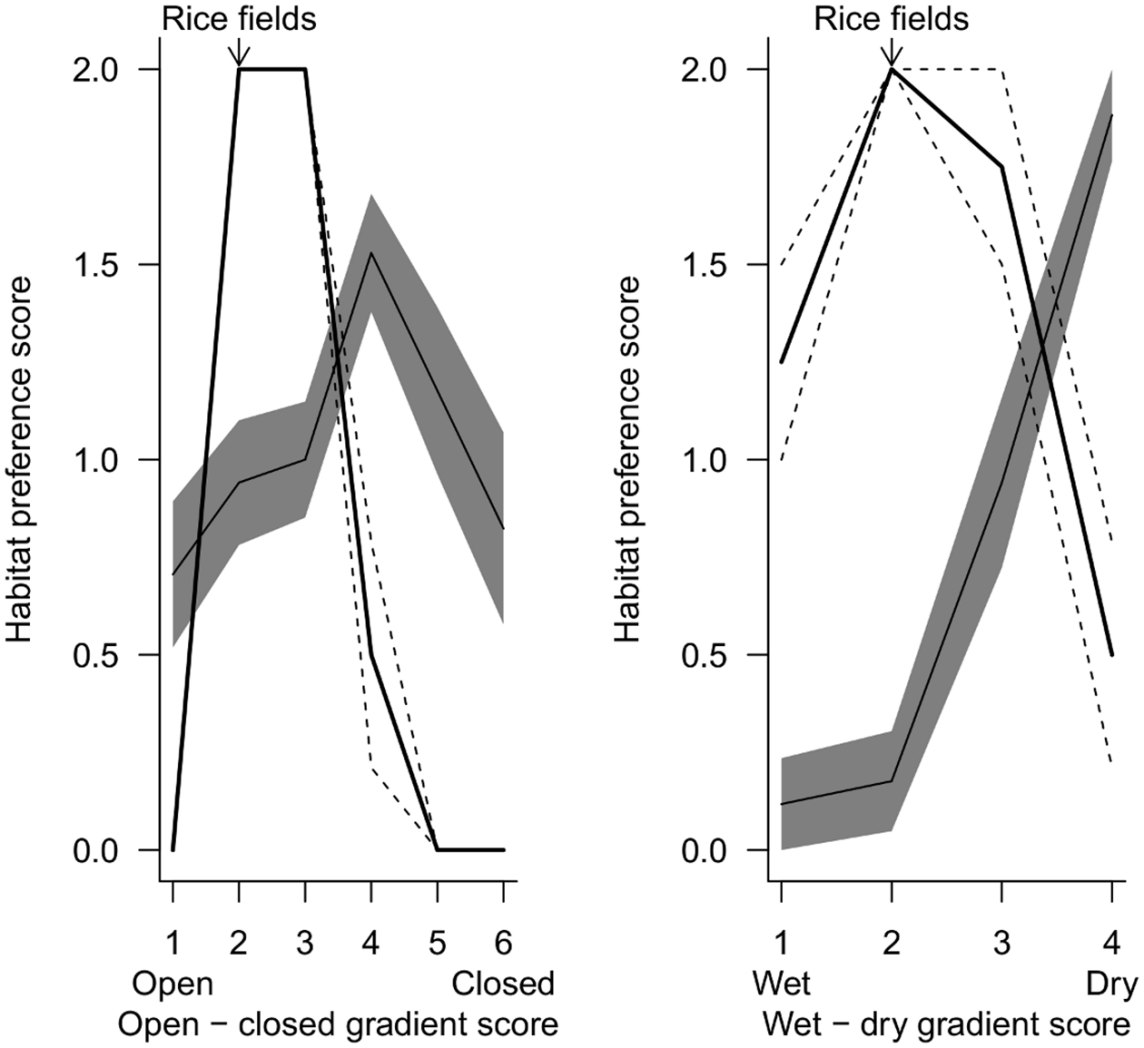
Habitat associations of native and non-native species along environmental gradients. These were from open to closed habitats (left) and wet to dry habitats (right). Mean habitat associations across non-native species are shown by a bold line, with dashed lines showing standard errors. Mean habitat associations across native species are shown by a solid line, with grey shading showing standard errors. Habitat associations for non-native species were based on descriptions of their habitat use in their native range.

Rice fields provided conditions that fell within the native-range habitat associations of the non-native species (Fig 5), and were positioned on both environmental gradients at the point with the greatest difference between the habitat associations of native and non-native species (Fig 5).

## Discussion

Rice fields are a recent human-modified land-use that we find to provide fewer resources for native seed-eating birds than adjacent open habitats. This was reflected by the lower functional diversity and species packing of native bird communities in rice fields. This scarcity of resources might be expected to hinder the ability of non-native seed-eating birds to colonise rice fields. However, non-native species were positively associated with rice fields, and plugged the missing functional diversity in rice fields, with differences in resource use allowing non-native species to exploit resources found in rice fields. These results are consistent with novel human modified land-uses providing resources that are underexploited by native species, and potentially facilitating the establishment of non-native species.

Although the non-native species in our study system were functionally similar to native species, subtle niche differences meant that non-native birds were better able to access the resources provided by rice fields. For example, most native seed-eating bird species primarily feed on forbs while the non-native species are lighter and more agile [23, 27, 28] and in this study were observed to feed extensively on flimsy grasses, which dominated rice field margins (as indicated by the lower forb species richness and lower plant weight holding capacity in rice field margins). These observations of resource use for feeding support previous work on these species [23, 30]. Differences in the selection of shelter habitat were also important for allowing non-native species to exploit rice fields, with native species tending to select trees and bushes for shelter, while non-native species selected emergent vegetation for shelter. Trees and bushes were widely available in wetlands and more traditional agricultural systems, either forming field boundaries in more open agricultural systems, being scattered amongst lower growing crops in cropping systems that mix fruit trees (e.g. olives) with herbaceous crops, or growing alongside rivers and natural wetlands. In rice fields, field boundaries were instead formed by emergent vegetation, with few trees and bushes to provide shelter. Thus rice fields contained suitable shelter habitat for non-native species, but shelter for native species was limited. This difference in shelter habitat selection is also reflected in breeding habitat selection, as the non-native species documented often nested in emergent vegetation [23] while many of the native species use trees and bushes for nesting [29], indicating that there are likely to be fewer nest sites for native species in rice fields than in other open habitats. Differences between native and non-native species in the selection of resources for shelter were more pronounced when selection was calculated using the availability of resources across all point counts, and therefore implicitly including occupancy effects (S1 Fig). This supports the role of the availability of shelter habitat in influencing occupancy patters.

We found that native bird communities that were species poor consisted of functionally dissimilar species; these communities were characteristic of rice fields. This pattern could arise if rice fields lack sufficient resources to support functionally similar native species, as functional overdispersion can result from the competitive exclusion of similar species [35]. Alternatively, this pattern could result if rice fields impose an environmental filter which only some functionally dissimilar generalists can pass through. Some support for the latter comes from similar densities of some native species, such as corn bunting *Emberiza calandra* and linnet *Carduelis cannabina*, and lower densities of other native species, such as serin and greenfinch, in rice fields compared to in natural wetlands and more traditional agriculture, indicating that some native species find rice fields more suitable than others.

The positive associations of non-native species with rice fields, negative associations of many native species with rice fields and differences in resource use that allow non-native species to better exploit resources in rice fields collectively demonstrate the *potential* for under-exploited resources to facilitate invasion by reducing the amount of competition non-native species experience. However, we cannot demonstrate that this is the causal mechanism underlying these patterns from our observational dataset. We therefore consider whether alternative mechanisms could have led to differences in the associations of native and non-native species with rice fields.

An alternative explanation to the lower density and functional diversity of native species in rice fields being due to environmental filtering is that the occurrence of native species in rice fields is reduced by competitive exclusion by non-native species. We cannot definitively test whether this has occurred with our observational dataset, however two lines of evidence suggest that it is unlikely. Firstly, we excluded the effects of occupancy when quantifying resource selection (by only assessing the availability of resources at point counts where a species was recorded), so our estimates of resource selection would not be affected by native species being excluded from locations by inter-specific competition. Thus compared to other open habitats, rice fields have fewer resources for native species, regardless of whether non-native species out-compete native species for these resources. Secondly, native species were on average heavier than non-native species, so we would expect them to be competitively dominant.

The differences in resource use between native and non-native species we document could, if sufficient, allow native and non-native species to avoid competition in the same location by using different resources. Both our data and previous studies indicate that the native species in our study system prefer feeding on forbs to grasses [29–30], while non-native species prefer feeding on grasses [27–28], supporting the potential operation of this mechanism. Despite this, the native birds in this study do feed on grasses (but to a lesser extent than on forbs) [29–30], indicating some overlap in resource use with non-native birds, and thus the potential for competition. The lower densities and functional diversity of native species in rice fields may be due to the additive effect of reduced feeding resources (fewer forbs, lower capacity of plants to hold the weight of heavy birds) and limited shelter rather than being due to differences in the amount of feeding resources alone.

An alternative but not mutually exclusive explanation to the presence of underexploited resources driving the association of non-native species is that the four non-native species in this study were positively associated with rice fields because they provide the conditions most similar to those occupied in their native range. The key question is thus whether these non-native species would still be primarily associated with rice fields in the absence of competition from native species in other habitats. It is not possible to definitively answer this question without experimental manipulations, which are not feasible in this system and at this scale. However, comparing habitat associations of these non-native species in their native range with their habitat associations in their non-native range can offer insight into the role of competition and niche-matching in driving these habitat associations. On both hydrological and open to closed gradients, rice fields lie at the point where non-native species have their most positive native-range habitat association, indicating that rice fields do provide a good match to the habitats occupied in the species’ native range. Such niche-matching has been widely documented in non-native species [41]. However, based on their native habitat associations these non-native species would also be expected to occur in natural wetlands, river valleys and heterogeneous and abandoned agriculture [28, 31]. While all these habitats are colonised to some extent in the Iberian Peninsula [23, 24, 42], the results of this study show that non-native species were more likely to be found in rice fields than in these other habitats. This supports the role of factors in addition to niche-matching in determining habitat selection by non-native species. Differences between the habitat associations of native and non-native species were greatest at the point in the environmental gradient where rice fields occur (Fig 5), indicating rice fields were potentially the habitat with the greatest amount of underexploited resources (i.e. resources that could not be exploited by native species) that could be accessed by non-native species.

Analysis of patterns of functional diversity using presence-absence data may have been affected by differences in the detectability of species between habitats. This was unlikely to have affected our results, as modelled estimates of detection probability revealed that there was a bias towards higher detection probabilities of native species in rice fields, when we found that the occurrence and densities of non-native species were higher in rice fields. Yellow-crowned bishops were the only non-native species more likely to be detected in rice fields than in other habitats. Again this was unlikely to have affected our results as, firstly, their densities (correcting for detectability) were significantly higher in rice fields than in other sites, and secondly, excluding them from analyses did not prevent non-native species filling the missing functional diversity in rice fields. This latter analysis is important, as it demonstrates that no single non-native species was responsible for plugging the gap in functional diversity in rice fields.

Our motivation for quantifying functional diversity, using traits related to resource use, was that it provides an indication of the diversity of resources being used by a bird community. Non-native birds fill the missing functional diversity in rice fields, indicating that rice fields can support a similar diversity of bird resource use to natural wetlands and more traditional agriculture despite the lower diversity of resource use by native species. Functional diversity can also be used to gain insight into ecosystem functioning, with functionally diverse bird communities potentially feeding on and dispersing a wider range of seeds, and to a lesser extent feeding on a wider range of invertebrates (as many species fed on invertebrates to a limited extent in the breeding season–S1 Appendix Table 2). In agricultural systems seed-eating birds can provide an ecosystem service by consuming weed seeds [43], however, birds also disperse seeds [44], so can also perform an ‘ecosystem disservice’ by dispersing weed seeds into crops. We did not aim to quantify the contribution of non-native species to ecosystem functioning, so it is uncertain whether plugging the gap in functional diversity in rice fields represents an important contribution to ecosystem functioning and ecosystem service provision.

Analyses in this study were restricted to a single guild, seed-eating birds, rather than the entire bird community. Other groups that occasionally eat seeds, such as larks (Alaudidae) and tits (Paridae), could compete with non-native seed-eating birds and reduce the degree to which resources in rice fields were underexploited. The extent to which this happened is likely to be limited, as differences in foraging mechanisms and bill morphology mean that these species are functionally distinct from the seed-eating guild considered here. For example, in a previous analysis tits have been found to occupy a functional space distinct to that of the seed-eating guild [45]. Crested larks *Galerida cristata* were present in most point counts but were never observed feeding in field margins, while tits were rarely recorded during our point counts. It is therefore unlikely that inclusion of these species would have affected our conclusions.

A previous study quantified the similarity of common waxbills to native species based on their resource use for feeding and nesting, habitat associations and morphology, and concluded that common waxbills occupied niche space peripheral to that occupied by native species [45]. Our work supports and substantially expands on this by demonstrating differences in resource use in other non-native birds, and by showing how these differences in resource use can enable non-native birds to exploit resources in a human-modified habitat that is underexploited by native species. Garcia et al. [46] demonstrated how generalist non-native birds were able to maintain frugivory networks following declines by native species, which combined with our observation that non-native species filled missing functional diversity in a human modified habitat suggests that non-native species can exploit vacant niche space. Our study provides two novel insights in addition to this, firstly demonstrating that this vacant niche space potentially facilitates colonisation by non-native species, and secondly showing how subtle differences in species’ ability to use resources can affect the degree to which they occur in anthropogenic habitats.

Conversion of natural and semi-natural habitats to human-modified ones has previously been found to reduce species richness [47], with a reduction in functional diversity due to local extinction of species with certain traits [48]. In addition, intensification of existing agriculture can alter the traits of plant communities to favour a few functional groups [49]. Our work extends this by highlighting potential mechanisms which lead to the functional diversity of a guild of native species being lower in a novel land-use than in more traditional human-modified land-uses. Human-modified habitats present a filter that only some species in a species pool are able to pass through [11]. We expect traditional land-uses that contain functionally similar habitat elements to natural habitats are likely to pose less of a habitat filter than more altered land-uses, but further work is needed to assess whether the novelty of human-modified habitats (compared to natural habitats) is an important driver of functional diversity loss in other systems. For this loss of functional diversity to result in increased invasibility we would expect human-modified habitats to have more underexploited resources. Some support for the increased invasibility of human-modified habitats is provided from observations by Maskell et al. [19] that non-native plant species richness in Great Britain is higher in human-modified habitats, including agricultural systems. However, in that study native and non-native species richness were positively correlated, so high non-native species richness may have resulted from greater provision of certain resources (e.g. through fertiliser addition) rather than the inability of native species to use available resources.

This study demonstrates that a novel land-use can provide resources that were underexploited by native species, but which could be exploited by a suite of non-native species. This has the potential to facilitate invasion by non-native species by reducing the amount of competition they experience. This suggestion is supported by the positive association of non-native species with rice fields. Further work is required to investigate whether the results presented here can be generalised to other systems. If novel human-modified habitats typically have underexploited resources, these may provide ‘welcome mats’ to establishing non-native species, and so should be targeted by surveillance programmes.

## Supporting Information

S1 Fig Resource selection using different definitions of resource availability. (DOCX)

S1 Table Estimated detectability of study species in rice fields and other habitats. (DOCX)

S2 Table Scores given to major habitat types on environmental gradients. (DOCX)

S3 Table Habitat association scores of study species. (DOCX)

S4 Table Trait data used to calculate functional diversity. (CSV)

S5 Table Point count data used in this analysis. (CSV)

S1 Appendix Methods for obtaining and scoring trait data, including field observations used to support trait scoring. (DOCX)

S2 Appendix Species packing in functional space in rice fields and other open habitats. (DOCX)
