# Supplementary material for: An Anthropogenic Habitat Facilitates the Establishment of Non-Native Birds by Providing Underexploited Resources
Source: PLoS One. 2015 Aug 14;10(8):e0135833. doi: 10.1371/journal.pone.0135833 (PMC4537089; doi:10.1371/journal.pone.0135833)
Supplement: S1 Fig — (DOCX) [file pone.0135833.s001.docx]

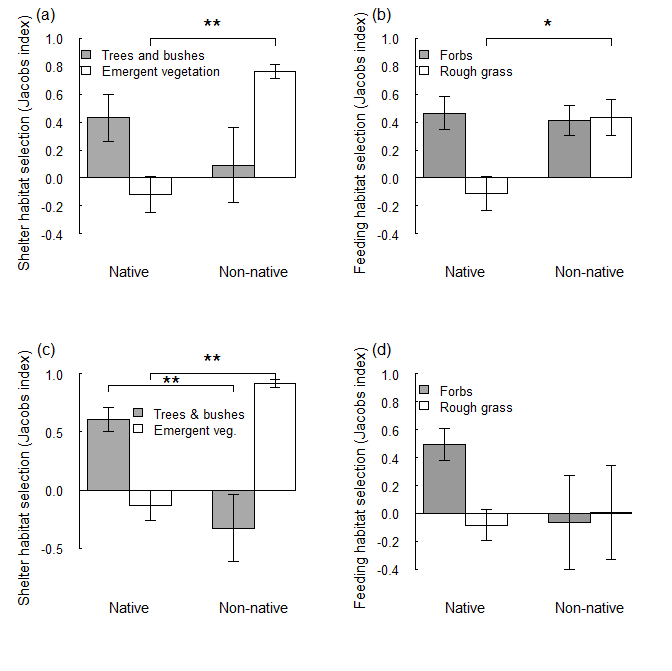


**Figure S1.** Selection of resources for shelter (a and c) and feeding (b and d). In (a) and (b) Jacobs index has been calculated using the availability of each resource in point counts were the species was recorded (i.e. same as Fig. 4 in main body), thus excluding effects of occupancy from the assessment of resource selection. In (c) and (d) Jacobs index has been calculated using resource availability across all point counts. Error bars show standard error. Wilcoxon-Mann-Whitney tests were used to test whether differences in resource selection of native and non-native species were significantly different, *** denotes *P*<0.001,** denotes *P*<0.01, * denotes P<0.05.
