# Supplementary material for: An Anthropogenic Habitat Facilitates the Establishment of Non-Native Birds by Providing Underexploited Resources
Source: PLoS One. 2015 Aug 14;10(8):e0135833. doi: 10.1371/journal.pone.0135833 (PMC4537089; doi:10.1371/journal.pone.0135833)
Supplement: S1 Table — (DOCX) [file pone.0135833.s002.docx]

**S1 Table**. **Estimated detectability of study species in rice fields and other open habitats.**

| Species | Detection probability ^a^ | | W ^b^ | P |
| --- | --- | --- | --- | --- |
|  | Other habitats | Rice fields |  |  |
| House Sparrow | 0.827 | 0.821 | 8293 | 0.163 |
| Serin | 0.652 | 0.703 | 1525 | 0.001 |
| Goldfinch | 0.741 | 0.740 | 3296.5 | 0.218 |
| Greenfinch | 0.769 | 0.789 | 859 | 0.001 |
| Linnet | 0.601 | 0.559 | 1122.5 | <0.001 |
| Corn Bunting | 0.999 | 0.998 | 1259 | 0.124 |
| Tree Sparrow | 0.609 | 0.732 | 39 | 0.017 |
| Common Waxbill | 0.647 | 0.588 | 5134.5 | <0.001 |
| Yellow-crowned Bishop | 0.453 | 0.630 | 9 | 0.196 |
| Black-headed Weaver | 0.603 | 0.592 | 31 | 0.680 |
| Red Avadavat | 0.544 | 0.782 | 7 | 0.001 |

^a^ Mean detection probabilities predicted by the best performing detection model for each species.

^b^ Wilcoxon-Mann-Whitney test of difference between habitats in predicted detectability for each species.
