# Supplementary material for: An Anthropogenic Habitat Facilitates the Establishment of Non-Native Birds by Providing Underexploited Resources
Source: PLoS One. 2015 Aug 14;10(8):e0135833. doi: 10.1371/journal.pone.0135833 (PMC4537089; doi:10.1371/journal.pone.0135833)
Supplement: S2 Table — (DOCX) [file pone.0135833.s003.docx]

**S2 Table.** Scores given to major habitat types on environmental gradients.

| Habitat type | Hydrological gradient | Open - closed gradient | Notes |
| --- | --- | --- | --- |
| Arable | 4 | 1 |  |
| Gardens | 4 | 4-5 | This is given 4 on the open-closed gradient unless gardens are described as wooded or well vegetation, when they are given 5 |
| Hedged field margins | 4 | 4 |  |
| Heterogeneous cultivation | 4 | 4 |  |
| Natural grassland | 4 | 1 |  |
| Non-woody riverine vegetation | 2 | 2 | This is given a lower hydrological gradient score than riverine scrub as this category includes riverine emergent vegetation |
| Open woodland | 4 | 5 |  |
| Orchards | 4 | 4 |  |
| Pasture | 4 | 1 |  |
| Rank grassland | 4 | 2 |  |
| Rice fields | 2 | 2 |  |
| Riverine scrub | 3 | 4-5 | This is given 4 on the open-closed gradient if it is described as light or open scrub, and 5 if it is described as dense, thick or heavy scrub |
| Savannah | 4 | 4-5 | This is given 4 or 5 on the open-closed gradient depending on tree cover |
| Scrub | 4 | 4-5 | This is given 4 on the open-closed gradient if it is described as light or open scrub, and 5 if it is described as dense, thick or heavy scrub |
| Wet grassland | 3 | 1 |  |
| Wet woodland | 3 | 6 |  |
| Wetlands | 1 | 3 |  |
| Woodland | 4 | 6 |  |
| Woodland edge | 4 | 5 |  |
| Wood-pasture/ Dehesa | 4 | 4-5 | This is given 4 or 5 on the open-closed gradient depending on tree cover |
