# Supplementary material for: An Anthropogenic Habitat Facilitates the Establishment of Non-Native Birds by Providing Underexploited Resources
Source: PLoS One. 2015 Aug 14;10(8):e0135833. doi: 10.1371/journal.pone.0135833 (PMC4537089; doi:10.1371/journal.pone.0135833)
Supplement: S3 Table — (DOCX) [file pone.0135833.s004.docx]

**S3 Table.** Habitat association scores of study species.

| Species | Gradient | Native | 1 | 2 | 3 | 4 | 5 | 6 |
| --- | --- | --- | --- | --- | --- | --- | --- | --- |
| Black-headed Weaver | Open - Closed | n | 0 | 2 | 2 | 0 | 0 | 0 |
| Black-headed Weaver | Hydrological | n | 2 | 2 | 1 | 0 |  |  |
| Bullfinch | Open - Closed | y | 0 | 0 | 0 | 1 | 2 | 2 |
| Bullfinch | Hydrological | y | 0 | 0 | 0 | 2 |  |  |
| Chaffinch | Open - Closed | y | 1 | 1 | 1 | 2 | 2 | 2 |
| Chaffinch | Hydrological | y | 0 | 0 | 1 | 2 |  |  |
| Cirl Bunting | Open - Closed | y | 0 | 1 | 1 | 2 | 1 | 0 |
| Cirl Bunting | Hydrological | y | 0 | 0 | 1 | 2 |  |  |
| Common Waxbill | Open - Closed | n | 0 | 2 | 2 | 1 | 0 | 0 |
| Common Waxbill | Open - Closed | n | 1 | 2 | 2 | 1 |  |  |
| Corn Bunting | Hydrological | y | 2 | 2 | 2 | 1 | 0 | 0 |
| Corn Bunting | Open - Closed | y | 0 | 0 | 2 | 2 |  |  |
| Crossbill | Hydrological | y | 0 | 0 | 0 | 0 | 1 | 2 |
| Crossbill | Open - Closed | y | 0 | 0 | 0 | 2 |  |  |
| Goldfinch | Hydrological | y | 0 | 1 | 1 | 2 | 2 | 0 |
| Goldfinch | Open - Closed | y | 0 | 0 | 2 | 2 |  |  |
| Greenfinch | Hydrological | y | 1 | 1 | 1 | 2 | 2 | 2 |
| Greenfinch | Open - Closed | y | 0 | 1 | 1 | 2 |  |  |
| Hawfinch | Open - Closed | y | 0 | 0 | 0 | 1 | 2 | 2 |
| Hawfinch | Hydrological | y | 0 | 0 | 2 | 2 |  |  |
| House Sparrow | Open - Closed | y | 2 | 2 | 2 | 1 | 0 | 0 |
| House Sparrow | Hydrological | y | 0 | 0 | 0 | 2 |  |  |
| Linnet | Open - Closed | y | 1 | 2 | 1 | 2 | 1 | 0 |
| Linnet | Hydrological | y | 0 | 0 | 1 | 2 |  |  |
| Ortolan Bunting | Open - Closed | y | 2 | 1 | 1 | 2 | 2 | 0 |
| Ortolan Bunting | Hydrological | y | 0 | 0 | 0 | 2 |  |  |
| Red Avadavat | Open - Closed | n | 0 | 2 | 2 | 1 | 0 | 0 |
| Red Avadavat | Open - Closed | n | 1 | 2 | 2 | 0 |  |  |
| Reed Bunting | Hydrological | y | 0 | 1 | 2 | 1 | 0 | 0 |
| Reed Bunting | Open - Closed | y | 2 | 2 | 2 | 0 |  |  |
| Serin | Hydrological | y | 1 | 1 | 1 | 2 | 2 | 2 |
| Serin | Open - Closed | y | 0 | 0 | 0 | 2 |  |  |
| Siskin | Hydrological | y | 0 | 0 | 1 | 1 | 2 | 2 |
| Siskin | Open - Closed | y | 0 | 0 | 2 | 2 |  |  |
| Spanish Sparrow | Hydrological | y | 1 | 1 | 1 | 2 | 0 | 0 |
| Spanish Sparrow | Open - Closed | y | 0 | 0 | 2 | 2 |  |  |
| Tree Sparrow | Open - Closed | y | 0 | 1 | 1 | 2 | 1 | 0 |
| Tree Sparrow | Hydrological | y | 0 | 0 | 0 | 2 |  |  |
| Yellow-crowned Bishop | Open - Closed | n | 0 | 2 | 2 | 0 | 0 | 0 |
| Yellow-crowned Bishop | Hydrological | n | 1 | 2 | 2 | 1 |  |  |
