# Supplementary material for: An Anthropogenic Habitat Facilitates the Establishment of Non-Native Birds by Providing Underexploited Resources
Source: PLoS One. 2015 Aug 14;10(8):e0135833. doi: 10.1371/journal.pone.0135833 (PMC4537089; doi:10.1371/journal.pone.0135833)
Supplement: S1 Appendix — (DOCX) [file pone.0135833.s007.docx]

**S1 Appendix. Methods for obtaining and scoring trait data, including field observations used to support trait scoring.**

Data on the diet, foraging behaviour, nesting habits and morphology of native species primarily came from [1, 2], supplemented by quantitative diet data from [3] for finches found in the UK (i.e. chaffinch, greenfinch, goldfinch, siskin, linnet, crossbill, bullfinch and hawfinch). Equivalent data for non-native species came from [4], supplemented with data from [5] for the common waxbill bill morphology and nesting period. We did find any published data on the diet of black-headed weavers or yellow-crowned bishops in their non-native range, so scored them for these traits based on field observations (Table S2.1), using literature references from their native range to aid scoring when field observations was scarce or absent [6-8].

**S1 Appendix Table 1.** Field observations used to support trait scoring.

| Species | Adult diet | Nestling diet | Agility |
| --- | --- | --- | --- |
| Black-headed weaver | 25 feeding observations where food item noted, 28% invertebrate, 72% seeds. Also one occasion feeding on reed stem, but not known if gleaning invertebrates or eating green material. | 7 feeding observations where food seen in bill, 86% insect, 14% seed, however seed thought to be considerably underestimated as young frequently fed from crop after adults feeding on grasses. | 76% of feeding observations on ground, but also frequently fed in low vegetation, usually clinging to vertical stems but sometimes also bent stems, clinging to multiple stems to feed. |
| Yellow-crowned bishop | 11 feeding observations where food item known – all seed. | Not observed. | Regularly observed feeding on bent stems or upside down. Flushed from the ground, but all quantified feeding observations in low vegetation. |

Quantitative data on diet was not available for all species, so qualitative descriptions also had to be used. Descriptions in literature sources were compared to the qualitative descriptors (Table S2.2). Rules used to convert quantitative and qualitative data into ordinal scores is given in Table S2.2.

**S1 Appendix Table 2.** Rules used to assign scores to traits.

| Trait | Code | Quantitative score | Qualitative descriptor |
| --- | --- | --- | --- |
| Diet | 0 | 0% | Never/ exceptionally recorded in diet |
|  | 1 | <5% | Occasional part of diet |
|  | 2 | 5 to 25% | Regular minor component of diet |
|  | 3 | 25-50% | Forms a substantial part of diet, but not primary component |
|  | 4 | 50-75% | Forms the majority of diet, but considerable use of other food indicated |
|  | 5 | >75% of diet | The dominant part of the diet. Other foods only form minority of diet. |
|  |  |  |  |
| Feeding agility | 0 |  | Never/ exceptionally recorded |
|  | 1 |  | Rarely recorded |
|  | 2 |  | Regularly recorded doing activity, but not a major feeding mode |
|  | 3 |  | Common feeding mode |
|  |  |  |  |
| Foraging height | 0 |  | Never or rarely recorded |
|  | 1 |  | Occasionally forages at this height |
|  | 2 |  | Regularly forages at this height, but not primary foraging height |
|  | 3 |  | Primary foraging height |
|  |  |  |  |
| Feeding habitat | 0 |  | Never or rarely used |
|  | 1 |  | Occasionally used |
|  | 2 |  | Important habitat, but not clearly dominant |
|  | 3 |  | Dominant habitat |
|  |  |  |  |
| Food plants | 0 |  | Never/ rarely used |
|  | 1 |  | Occasionally used |
|  | 2 |  | Important food plant, but not clearly dominant |
|  | 3 |  | Dominant food plant |
|  |  |  |  |
| Nest location | 0 |  | Never/ rarely used |
|  | 1 |  | Sometimes used, but not primary nest location |
|  | 2 |  | Main nest location |
|  |  |  |  |
| Nest height | 0 | <1m |  |
|  | 1 | >1m |  |
|  |  |  |  |
| Nesting season | 0 |  | Never/ rarely recorded |
|  | 1 |  | Some individuals likely to be nesting, but not majority of the population |
|  | 2 |  | Main season |

**References for S1 Appendix**

1. Cramp S, Perrins C. Handbook of the Birds of Europe, the Middle East and North Africa: Crows to Finches. Oxford: Oxford University Press; 1994.

2. Cramp S, Perrins C. Handbook of the Birds of Europe, the Middle East and North Africa: Buntings to New World Warblers. Oxford: Oxford University Press; 1994.

3. Newton I. The adaptive radiation and feeding ecology of some British finches. Ibis. 1967;109(1):33-96. doi: 10.1111/j.1474-919X.1967.tb00005.x.

4. Matias R. Aves exóticas que nidificam em Portugal continental. Lisbon: ICNB; 2002.

5. Batalha HR, Ramos JA, Cardoso GC. A successful avian invasion occupies a marginal ecological niche. Acta Oecologica. 2013;49(0):92-8. doi: <http://dx.doi.org/10.1016/j.actao.2013.03.003>.

6. del Hoyo J, Elliot A, Christie D, editors. Handbook of the Birds of the World: Bush Shrikes to Old World Sparrows. Barcelona: Lynx Edicions; 2009.

7. del Hoyo J, Elliot A, Christie D. Handbook of the Birds of the World: Weavers to New World Warblers. Barcelona: Lynx Edicions; 2010.

8. Barnard P. Common Waxbill. In: Harrison JA, Allan DG, Underhill LG, Herremans M, Tree AJ, Parker V, et al., editors. The Atlas of Southern African Birds. 2: Passerines. Blairgowrie, South Africa: BirdLife South Africa; 1997. p. 612-3.
