# Supplementary material for: An Anthropogenic Habitat Facilitates the Establishment of Non-Native Birds by Providing Underexploited Resources
Source: PLoS One. 2015 Aug 14;10(8):e0135833. doi: 10.1371/journal.pone.0135833 (PMC4537089; doi:10.1371/journal.pone.0135833)
Supplement: S2 Appendix — (DOCX) [file pone.0135833.s008.docx]

**S2 Appendix.** Species packing in functional space in rice fields and other open habitats.


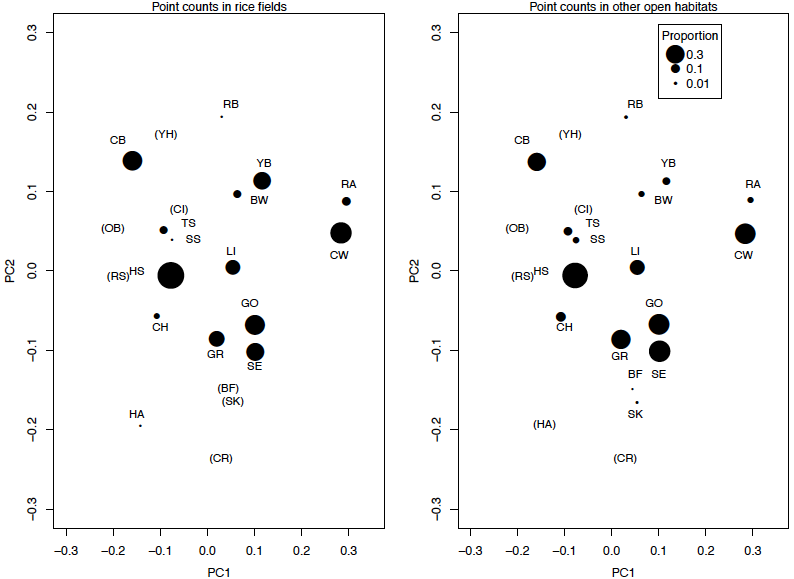


**S2 Appendix Figure 1.** **Species packing in rice fields and other open habitats.** Principal coordinates analysis was used to position species in two-dimensional space, where distances between species reflect Gower distances based on functional traits. Based on the correlation between species traits and principal coordinates axes scores, the first principal coordinates axes (PC1) was primarily related to body size (wing length *r* = -0.81, tarsus length *r* = -0.69, body mass *r* = -0.67), nestling diet (invertebrates *r* = -0.78) and feeding agility (ability to feed on bent stems *r* = 0.81). The second principal coordinates axes (PC2) was primarily related to food plants (grasses *r* = 0.87), feeding habitat (trees *r* = -0.80), nest height (height of nest site *r* = -0.77) and feeding height (tree layer *r* = -0.76). The size of each circle is proportional to the square root of the proportion of point count locations in which they were recorded (see legend). Species codes are shown next to circles. Species codes in parentheses denote species not recorded at any point count.

**S2 Appendix Table 1.** Species codes used in S2 Appendix Fig. 1.

| Species | Scientific name | Species code |
| --- | --- | --- |
| Black-headed weaver | *Ploceous melanocephalus* | BH |
| Bullfinch | *Pyrrhula pyrrhula* | BF |
| Chaffinch | *Fringilla coelebs* | CH |
| Common waxbill | *Estrilda astrild* | CW |
| Common crossbill | *Loxia curvirostra* | CR |
| Corn bunting | *Emberiza calandra* | CB |
| Goldfinch | *Carduelis carduelis* | GO |
| Greenfinch | *Carduelis chloris* | GR |
| Hawfinch | *Coccothraustes coccothraustes* | HA |
| House sparrow | *Passer domesticus* | HS |
| Linnet | *Carduelis cannabina* | LI |
| Ortolan bunting | *Emberiza hortulana* | OB |
| Red avadavat | *Amandava amandava* | RA |
| Reed bunting | *Emberiza schoeniclus* | RB |
| Rock sparrow | *Petronia petronia* | RS |
| Serin | *Serinus serinus* | SE |
| Siskin | *Carduelis spinus* | SK |
| Spanish sparrow | *Passer hispaniolensis* | SS |
| Tree sparrow | *Passer montanus* | TS |
| Yellow-crowned bishop | *Euplectes afer* | YB |
| Yellowhammer | *Emberiza citrinella* | YH |
